# A conversation with Kevin Gibbs, MD, associate professor, Wake Forest University School of Medicine

**DOI:** 10.1017/cts.2025.10135

**Published:** 2025-09-10

**Affiliations:** Clinical Research Forum, Washington, DC, USA

## Top 10 clinical research achievement awards Q & A

This article is part of a series of interviews with recipients of the Clinical Research Forum’s Top 10 Clinical Research Achievement Awards. This interview is with Kevin Gibbs, MD, Associate Professor, Wake Forest University School of Medicine. Dr Gibbs’s research focuses on improving outcomes for patients with critical illness who require life support. He received a 2025 Top 10 Clinical Research Achievement Award for “Noninvasive Ventilation for Preoxygenation During Emergency Intubation.” *The interview has been edited for length and clarity.*

## What drew you to the field of clinical research?

As a clinician, one of the things that I find most frustrating is being confronted with medical decisions where there are multiple acceptable treatment regimens available and having to choose one, often without knowing which is the most effective. It’s a scenario that happens to practitioners all the time, and when it does, we have to make choices based on our specialties, on our individual experiences as doctors, or on institutional cultures. I think we owe it to our patients to try and determine which interventions are the best, and that has been a driver for me throughout my career.

## The award-winning trial was precisely this kind of comparative effectiveness study

Yes. Our network, the Pragmatic Critical Care Research Group, has spent the last 10 or so years focused on comparing treatments for patients who require life support in the form of a breathing tube and a breathing machine. This trial investigated how preoxygenation with noninvasive ventilation, as compared with preoxygenation with an oxygen mask, affects the concentration of oxygen in the blood during tracheal intubation.

## Why is preoxygenation so important during intubation?

More than one million patients undergo tracheal intubation each year in the USA. The most common complication during the procedure is low oxygen levels in the blood (hypoxemia), which is associated with worse outcomes. Because of that, there’s been a lot of interest in the best ways to prevent hypoxemia during this procedure. We know that preoxygenation decreases the risk of hypoxemia, and most critically ill adults receive preoxygenation by means of a loose-fitting oxygen mask. Some clinicians have advocated preoxygenation with noninvasive ventilation, which uses a tight-fitting mask and pressure to help the patient breathe as an alternative to an oxygen mask. With this trial, we wanted to find out which method of preoxygenation is better to prevent hypoxemia.

## What did the results show?

Among the 1301 patients enrolled in the trial, we found that preoxygenation with noninvasive ventilation reduced hypoxemia during intubation compared to preoxygenation with an oxygen mask. Even for patients whom clinicians think are at low risk of complication, we found that using noninvasive ventilation is better.

## Where is this research heading next?

I, along with the entire Pragmatic Critical Care Research Group, remain committed to identifying the safest ways to place people on life support. We are nearing completion of our next trial, which will help answer which of the commonly used medications is best to sedate patients for intubation. In addition, we are launching another trial comparing the effectiveness of smaller versus larger breathing tubes for mechanical ventilation for critically ill adults. I’d also like to add that we are interested in applying these techniques beyond emergency tracheal intubation in the critical care space. As I mentioned at the start of this interview, every day, clinicians are confronted by situations where there are multiple therapeutic options, but no clear evidence to guide them. We need to do better, and identifying questions that enable us to perform these comparative effectiveness studies safely and ethically is really important.

## What role does team science play in this research?

I think all science is team science, but these trials in particular are truly a team science endeavor. This study, for example, was performed at 17 ICUs and seven emergency departments across the country. There was a tremendous amount of feedback on trial design from key stakeholders, including emergency medicine physicians, ICU physicians, and patient engagement groups, and we were funded by the US Department of Defense. All these different parts came together, and from startup to completion, this trial was completed in about a year.

## That’s a fast timeline for clinical research. What do you attribute that to?

Honestly, it is largely driven by the passion of the investigators trying to improve this procedure. For years, this group has done this work in a mostly unfunded way – because they are so passionate about trying to improve the care for patients undergoing these life-threatening procedures. As it turns out, this was one of the first trials that had federal funding, which helped speed up the timeline, because it was a priority for them, too. Lastly, with this type of research, when we apply existing standard of care treatments and compare them, we can have less lead-in time than there are for studies exploring novel treatments. It’s really satisfying to get an answer in a relatively short amount of time.

## What advice do you have for people starting careers in clinical research?

Scientific careers are frequently nonlinear, so my first piece of advice is to be open to opportunities that emerge, despite any preconceived notions of what your specialization is going to be. I wasn’t intending to be an airway researcher at the beginning of my career. I started in basic science, researching T cell biology. I enjoyed that, but I found it was too far removed from patient care, and I became increasingly interested in clinical research, making career moves in that direction. My other piece of advice is that, while you have to be passionate about the project you’re embarking on when you’re in training, you also need to be aware that the project is not necessarily the most important thing. The most important thing is developing the skill sets that will enable you to succeed as an independent investigator later. You need to emerge from your training with skills that are translatable and can be applied to different questions over time.

## How have you been able to stay open-minded about new opportunities as they arose during your career?

One of my guiding career principles is that I have been passionate about science – in whatever form – for as long as I’ve been doing it. So, yes, researchers need to be enthusiastic about their specific projects. But it’s more important, at least in my experience, to be passionate about the scientific method. Projects come and go, but if you’re committed to science, there is always work to be done.

## Outside of clinical research, what other activities do you enjoy?

I’m a native plant gardener, and I’m working to turn my front yard into a pocket-sized prairie. I find being outside is calming, and it’s been gratifying to see the garden evolve over time. Also, I recently took up indoor bouldering (a form of rock climbing performed on artificial walls), which is a completely different kind of outlet. It’s enjoyable because I like having a low-stakes problem to solve while I’m exercising, and it’s a good break from the work that I do.

